# A cluster randomized trial of standard quality improvement versus patient-centered interventions to enhance depression care for African Americans in the primary care setting: study protocol NCT00243425

**DOI:** 10.1186/1748-5908-5-18

**Published:** 2010-02-23

**Authors:** Lisa A Cooper, Daniel E Ford, Bri K Ghods, Debra L Roter, Annelle B Primm, Susan M Larson, James M Gill, Gary J Noronha, Elias K Shaya, Nae-Yuh Wang

**Affiliations:** 1 Welch Center for Prevention, Epidemiology, and Clinical Research, Johns Hopkins University, Baltimore, Maryland, USA; 2 Department of Medicine, Johns Hopkins University School of Medicine, Baltimore, Maryland, USA; 3 Department of Epidemiology, Johns Hopkins Bloomberg School of Public Health, Baltimore, Maryland, USA; 4 Department of Health, Policy and Management, Johns Hopkins Bloomberg School of Public Health, Baltimore, Maryland, USA; 5 Department of Health, Behavior, and Society, Johns Hopkins Bloomberg School of Public Health, Baltimore, Maryland, USA; 6 American Psychiatric Association, Arlington, Virginia, USA; 7 Delaware Valley Outcomes Research, Newark, Delaware, USA; 8 Department of Family and Community Medicine, Jefferson Medical College, Philadelphia, Pennsylvania, USA; 9 Johns Hopkins Community Physicians, Baltimore Maryland, USA; 10 Department of Psychiatry, Good Samaritan Hospital, Baltimore, Maryland, USA; 11 Department of Biostatistics, Johns Hopkins Bloomberg School of Public Health, Baltimore, Maryland, USA

## Abstract

**Background:**

Several studies document disparities in access to care and quality of care for depression for African Americans. Research suggests that patient attitudes and clinician communication behaviors may contribute to these disparities. Evidence links patient-centered care to improvements in mental health outcomes; therefore, quality improvement interventions that enhance this dimension of care are promising strategies to improve treatment and outcomes of depression among African Americans. This paper describes the design of the BRIDGE (Blacks Receiving Interventions for Depression and Gaining Empowerment) Study. The goal of the study is to compare the effectiveness of two interventions for African-American patients with depression--a standard quality improvement program and a patient-centered quality improvement program. The main hypothesis is that patients in the patient-centered group will have a greater reduction in their depression symptoms, higher rates of depression remission, and greater improvements in mental health functioning at six, twelve, and eighteen months than patients in the standard group. The study also examines patient ratings of care and receipt of guideline-concordant treatment for depression.

**Methods/Design:**

A total of 36 primary care clinicians and 132 of their African-American patients with major depressive disorder were recruited into a cluster randomized trial. The study uses intent-to-treat analyses to compare the effectiveness of standard quality improvement interventions (academic detailing about depression guidelines for clinicians and disease-oriented care management for their patients) and patient-centered quality improvement interventions (communication skills training to enhance participatory decision-making for clinicians and care management focused on explanatory models, socio-cultural barriers, and treatment preferences for their patients) for improving outcomes over 12 months of follow-up.

**Discussion:**

The BRIDGE Study includes clinicians and African-American patients in under-resourced community-based practices who have not been well-represented in clinical trials to improve depression care. The patient-centered and culturally targeted approach to depression care is a relatively new one that has not been tested in most previous studies. The study will provide evidence about whether patient-centered accommodations improve quality of care and outcomes to a greater extent than standard quality improvement strategies for African Americans with depression.

**Trial Registration:**

ClinicalTrials.gov NCT00243425

## Background

In the United States, the majority of individuals with mental disorders are untreated or poorly treated, and this is particularly true for ethnic minorities [1,2]. Despite the proven efficacy of pharmacotherapy and psychotherapy for depression, several studies have found that African Americans with depressive disorders receive lower quality of care [3-5]. African Americans are more likely to seek mental health care in primary care settings [6], where disparities in diagnosis [4,7] and appropriate pharmacotherapy and psychotherapy referrals persist [3,4,8,9]. Disparities between African Americans and whites in the adequacy of depression treatment are related to lower rates of initiating treatment with antidepressant medications, and not to disparities in initiating counseling or receiving adequate medications or counseling once initiated[10,11]. Additionally, disparities in depression care are not entirely explained by differences in education, income, and health insurance coverage [5,12,13].

Physician knowledge, attitudes, and skills, patient-physician communication, as well as patient cultural beliefs, attitudes, and preferences, are potential intervention targets to improve outcomes and reduce disparities in depression care. Primary care physicians discuss depression at lower rates and engage in less rapport-building with African-American patients [14]; this could partially explain lower recognition and treatment rates among African-American patients. Compared to white patients, African-American patients express stronger preferences for counseling [15] and spiritual approaches [16], and more negative attitudes toward antidepressant medication [17,18], the most common form of treatment of depression used by primary care physicians.

Although quality improvement (QI) strategies for depression enhance appropriate care and improve clinical outcomes among minorities, some of these interventions are less effective among minorities than whites at improving functional outcomes, and they do not eliminate disparities in depression treatment and clinical outcomes over 12 months [19,20]. The Institute of Medicine includes patient-centeredness--'providing care that is respectful of and responsive to individual patient preferences, needs, and values, and ensuring that patient values guide all clinical decisions'--as an important dimension of healthcare quality [21]. Interventions that focus on patient-centered communication have shown improvements in patient adherence, patient satisfaction, and mental health outcomes [22]. Additionally, interventions that use cultural leveraging increase patients' knowledge, decrease access barriers, and improve providers' cultural competence [23]. However, with a few exceptions [19,24], most QI strategies for depression target provider knowledge of treatment guidelines and disease-oriented collaborative case management of patients, rather than the quality of patient-clinician communication or the cultural acceptability of treatment approaches for patients [25-28]. Improving outcomes for ethnic minority patients with depression beyond those achieved in standard QI interventions may require interventions aimed at improving patient-clinician relationships and making healthcare systems more responsive to patients' needs and preferences.

## Methods

### Study design and specific aims

#### Specific aim one

**Figure 1:**
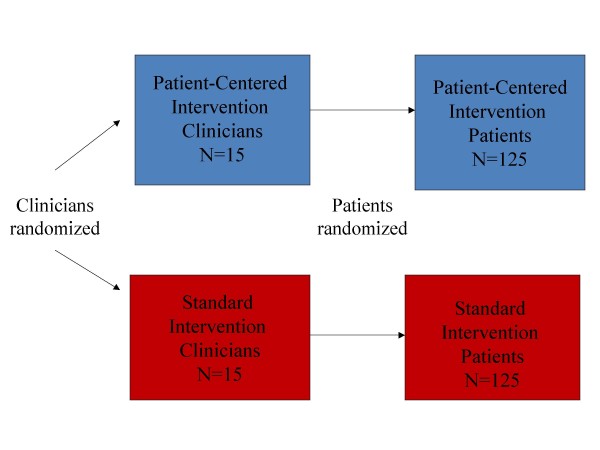
**BRIDGE study design**.

Recruit 30 primary care clinicians and 250 of their African-American patients with major depressive disorder (MDD) into the Patient-Centered Depression Care for African Americans study. The study has an acronym, 'BRIDGE'--Black Receiving Interventions for Depression and Gaining Empowerment. BRIDGE is a cluster randomized controlled trial with two experimental groups: a standard state-of-the-art depression intervention based on the chronic care model and a patient-centered intervention that supplements standard interventions for depression by tailoring them to individual patients' stated concerns and incorporating patient-centered communication skills and cultural sensitivity training for clinicians (Figure 1).

#### Specific aim two

Compare the effectiveness of the patient-centered intervention with the effectiveness of the standard intervention by evaluating their impact on the following patient outcomes at six and 12 months: depression symptom reduction; depression remission; functional status improvement; and receipt of guideline-concordant treatment.

#### Specific aim three

Compare the effectiveness of the patient-centered intervention with the effectiveness of the standard intervention by evaluating their impact on the following processes of care rated by patients at six and 12 months of follow-up: satisfaction with the quality of technical and interpersonal aspects of care in general; satisfaction with depression care in particular, and patients' and clinicians' attitudes regarding care of depression.

Interventions that target providers or patients alone have been insufficient to improve quality of depression care; therefore, it is important to test combined patient and clinician interventions. When interventions that target clinicians are initiated while the effects of the outcomes are measured at the patient level, a traditional randomized trial design with one patient per provider would offer optimal internal validity, but is often not feasible because it requires randomizing a large number of providers. Additionally, although further randomizing patients within each clinician cluster is theoretically possible and offers the added benefit of examining clinician and patient intervention effects separately, it is logistically challenging, resource intensive, and presents the relatively small possibility of cross contamination of the patient intervention among patients within the same clinician cluster. Therefore, for this study, the investigators chose a cluster design in which clinicians and their patients were randomized as one unit.

We hypothesize that compared to patients in the standard intervention group, at follow-up, patients in the patient-centered intervention group will decrease their level of depressive symptoms, remit from depression, improve their functional status, and receive guideline-concordant treatment to a greater extent; give higher ratings of partnership with providers, quality of depression care, and satisfaction; and report more positive attitudes regarding treatment choices for depression.

#### Study populations and settings

The BRIDGE study occurs in urban primary care sites in Baltimore, Maryland and Wilmington and Newark, Delaware. Maryland primary care sites are affiliated with Baltimore Medical System (one site), Baltimore Medical Surgical Associates, an affiliate of Greater Baltimore Medical Center (one site), Johns Hopkins Community Physicians (five sites), and Sinai Hospital, a member of MedStar Health (one site). Delaware primary care sites include two federally-qualified community health centers--Henrietta Johnson Medical Center (one site) and Westside Family Health (one site). These sites were chosen because they are community-based, serve a patient population that is at least 50% African American, include patients with a range of socioeconomic backgrounds, and are interested in improving care for their African-American patients.

### Recruitment strategies

#### Clinicians

Clinicians received an introductory letter that described the study and was co-signed by the medical director of their respective organization and the principal investigator. The letter outlined the goals of the study, gave a general description of the interventions, and described the responsibilities of clinicians caring for study patients. The letter also informed clinicians they would receive continuing medical education (CME) credits; an educational program about depression delivered to them in their office by a primary care physician/psychiatrist team; tailored, individualized feedback regarding their interviewing skills (either during or at the end of the study), and $200 paid to them or their organization. Subsequently, the principal investigator explained the study to prospective clinician participants at regular practice/staff meetings and answered any questions they had. At the conclusion of the presentation, clinicians were given a sign-up form that they could return immediately, fax, or mail to the principal investigator's office. Research staff made follow-up phone calls or visits to sites that had additional questions and to clinicians who did not respond within two weeks of a site presentation. Practice leaders facilitated communication with the clinicians and their staff.

#### Patients

On onsite recruitment days, participating clinics placed signs in registration areas that asked, 'Do you want to improve your emotional health and possibly earn up to $75?' The signs also specified which clinicians were participating in the study. During registration, patients were given a card that informed them their clinician was participating in a study to improve emotional health in African Americans, and they might be approached by a research assistant to ask if they were willing to participate. The patient could return the card to the receptionist if the patient was not African-American and/or did not wish to be approached. Those who agreed to be screened completed the procedures in a private area of the clinic. The research assistant described the study and obtained verbal consent to complete a 10-minute depression screening interview in a private room. Patients who were positive on the first-stage depression screen completed full written study consent, and a research assistant arranged for the patient's medical visit to be audio taped.

Patients who screened positive for depressive symptoms in clinical sites were called at home within two weeks of their onsite screening to complete the second-stage screen (to determine whether they had a diagnosis of MDD by the Composite International Diagnostic Interview, or CIDI) with a 40-minute telephone baseline interview [29,30]. If they met diagnostic criteria, they were randomized according to their clinician's intervention assignment and told to expect a phone call from a depression care manager within a few days; interviewers also obtained respondents' consent to be called for 30-minute follow-up interviews at 6, 12, and 18 months from the baseline interview.

Two other recruitment strategies supplemented the onsite approach. For these patient recruitment approaches, we obtained a waiver of HIPAA privacy authorization from the Johns Hopkins IRB and entered into agreements with the participating health plans to allow data sharing. The second recruitment approach was to have clinical managers at participating primary care sites help identify potential African-American study subjects using administrative and scheduling data. Field research staff then did onsite recruitment and screening at clinics where African-American patients were already scheduled to see a participating provider. The third patient recruitment approach was developed to meet requests from many participating clinicians that the study include some of their patients with known depression. Clinicians were asked to nominate 20 of their known African-American patient cases with MDD for possible inclusion in the study. Clinicians recommended patients with a broad spectrum of depression symptoms and in various stages of care. We sought and obtained IRB approval for clinicians to give research assistants patient contact information (*i.e*., name, address, phone number, and medical record number) for the purpose of preparing a letter that would be signed by the patient's clinician. Information packets containing the clinician's signed letter detailing the study goals and objectives and a prepaid study postcard that offered patients an option to refuse participation were sent to potential participants. Upon receipt of the letter, patients either called the study office, or, if no refusal postcard was received within two weeks of the mailing, they were called by study staff. In either case, during the telephone call patients were told about the study, asked questions to confirm eligibility, and asked if they would be willing to speak further about the study with a member of the study staff when they arrived at their next appointment. If they agreed, a field research assistant met the patient at the clinic prior to their next appointment. The research assistant administered the brief screening questionnaire, and the recruitment process proceeded as described above.

#### Eligibility criteria

Clinicians recruited for the BRIDGE study were general internists, family physicians, and nurse practitioners who saw patients at least 20 hours per week at one of the participating study sites. The clinicians were recruited without regard to race, gender, or age. Patients had to be between the ages of 18 and 75 years and report their race or ethnicity as African American; they had to be positive on a screener for major depressive and dysthymic disorder from the CIDI [30], which identified individuals who reported two weeks or more during the last year and one week or more during the past month when they felt sad, empty, depressed, or lost interest in things they normally enjoyed. The screener was self-administered with assistance as needed at the time of the recruitment visit. In addition, screen-positive patients had to meet criteria for one-year major depression on a subsequent structured interview [30], defined as: meeting DSM-IV criteria for MDD in the past year [31]and having symptoms present for at least one week in the past month, to be considered eligible for the study. Patients were excluded if they had an acute life-threatening condition or cognitive impairment that prevented them from completing the screener; indicated they did not intend to receive care in the clinic on an ongoing basis; had no access to a telephone; were currently pregnant, breastfeeding, or less than three months postpartum; screened positive for current bereavement, lifetime mania, or current alcohol or drug abuse; did not speak English; were currently receiving specialty mental health care; or reported immigrating to the United States within the preceding five years.

#### Randomization

Randomization was stratified by study site and conducted at the clinician level with patients sequentially selected within each randomized clinician (10 patients from each clinician). Within each study site (stratum), a randomization schedule was generated through computer by the study statistician using the Moses and Oakford algorithm [32]with the block sizes of two or four randomly created in a four to one ratio. Informed consent and baseline data collection from clinicians (background questionnaire and videotaped interview with the standardized patient) were completed before participating clinicians were randomly assigned (using a blind and secure allocation by computer) to either the standard or the patient-centered intervention. At the screening visit, patients of enrolled clinicians provided oral consent to complete the screening questionnaire. Once eligibility was confirmed, written consent and screening visit data were collected. Additionally, study staff obtained permission from eligible patients to contact them several times over the next 12 months. Patients were then assigned to the standard or patient-centered intervention according to their clinicians' randomization status. The patient recruiters were blinded to the clinicians' randomization assignment during recruitment, and the patients did not know their assigned treatment status until after enrollment. Due to the behavioral nature of the interventions, blinding or masking of study participants, investigators, and depression care managers was not possible. However, interviewers who collected baseline and follow-up data from patients at six and 12 months were masked to clinician and patient intervention assignment.

### Interventions

#### Clinician interventions

The clinician interventions were developed on a model previously shown to improve treatment knowledge for primary care clinicians [33,34]. Over the course of the study's 12-month clinician intervention period, clinicians in both intervention groups received two academic detailing visits for CME credit on the clinical management of depression from a team consisting of a primary care physician and a consultation-liaison psychiatrist. The visits were delivered within a two-month period, on average, for each clinician and focused on the latest advances in depression diagnosis and assessment (visit one) as well as treatment and referral (visit two), using the MacArthur Initiative on Depression and Primary Care Toolkit for clinicians [35]. The team visited clinicians individually or as a group according to the collective preference of clinicians at each site. In addition to approximately two hours of formal training, all clinicians received a monthly newsletter with study updates and summaries of recent journal articles related to depression. Each clinician who faced difficulty with patient diagnosis, motivation to initiate treatment, adherence to treatment, or response to treatment was invited to contact his or her consultation-liaison psychiatrist. Consultative services via monthly case conferences and collaborative patient visits were offered by the study psychiatrists; however, primary care clinicians chose to contact the consultants by telephone instead.

In contrast to the more didactic format used in the standard clinician intervention, the primary care physician/psychiatrist team that delivered academic detailing to clinicians in the patient-centered intervention guided each clinician through his or her personal interactive communication skills training program in a one-on-one or small group format with other clinicians in the practice who were also assigned to this intervention. The program featured a CD-ROM containing the clinician's interview with a simulated patient at baseline, fully-analyzed using the Roter Interaction Analysis System (RIAS) [36,37]. This sophisticated coding software generated tabular and graphic analyses of the verbal components of the interview (*e.g*., functional categories of communication and proficiencies useful in improving patient involvement in decision-making about depression treatment). Clinicians were also presented with the overall ratio of clinician to patient talk as a measure of verbal dominance [38]. The RIAS is based on a four-function model of medical interviewing that includes data-gathering, rapport-building, patient education and counseling, and facilitation and patient activation [39]. There were also targeted proficiencies related to depression included in the training program: recognizing depression; evaluating patients for associated conditions and suicidal ideation; assessing functioning and coping strategies, knowledge and beliefs about depression, and treatment preferences and concerns; and eliciting a commitment to the therapeutic plan. After viewing the analysis of communication by category, the software enabled clinicians to efficiently review examples of each function and proficiency within the interview. The CD-ROM also featured a video-glossary of simulated interviews illustrating the communication skills corresponding to each category. A companion workbook introduced clinicians to the RIAS and guided them through case-based exercises for skill practice.

#### Patient interventions

The standard and patient-centered patient interventions were both designed to incorporate state-of-the-art strategies that have been proven to enhance the quality of care for depression in primary care settings. Each intervention involved extensive one-on-one follow-up with a Depression Case Manager (DCM) to assess patients' depression status and to encourage adherence to recommended treatments and exposure to educational materials. Both DCMs were social workers with clinical experience; the standard DCM was a Caucasian woman, and the patient-centered DCM was an African-American woman.

The format and schedule for DCM contact were standardized across both interventions. Enrolled patients were contacted by their DCM within a week of the baseline telephone interview to schedule a 45-minute telephone conversation. Follow-up calls at one week, two weeks, and four to six weeks assessed each patient's depression level measured by the Patient Health Questionnaire (PHQ-9) score [40]. Symptomatic patients in the acute phase received weekly calls, while asymptomatic patients in the continuation and maintenance phases were called every eight and 12 weeks, respectively. Each DCM was also available to her patients by telephone as needed throughout the duration of the 12-month intervention.

The common goals of the standard and patient-centered DCMs were to provide needs assessment (five core areas for the standard DCM, 11 core areas for the patient-centered DCM), education, and activation messages to patients (Table 1). In addition to core areas, the needs assessment touched on active coping strategies, social stressors, and social support. To increase the efficiency and effectiveness of depression care, during every follow-up call each DCM monitored symptoms, functional status, and general health of her patients. In addition, each DCM reviewed the psychotherapy, medication, and/or alternative treatment plan developed by the patient and his or her primary care clinician. A report of relevant information was prepared for the patient, primary care clinician, and/or mental health specialist after each contact.

**Table 1:** Features of the BRIDGE study patient intervention

Data collected/delivered	Standard intervention	Patient-centered intervention
Needs assessment (five core assessment areas)	X	

Patient-centered needs assessment^1 ^(11 core assessment areas)[41]		X

Education and Activation	X	X

Social support/informal counseling	X	X

Standard education materials	X	

Culturally targeted education materials		X

Black mental health alliance resource list		X

Cultural information packet for MH Providers		X

^1^More in-depth individualized questioning (using Kleinman's explanatory model approach [41]) about symptoms, functional status, social support, treatment preferences compared to the standard needs assessment. Additional assessment questionswere about literacy and language, spirituality, financial concerns, and clinician relationship.

In contrast to the standard DCM, the patient-centered DCM conducted a more comprehensive needs assessment that explored the meaning of illness from the patient's perspective [41], his or her use of spirituality as an active coping strategy, and social stressors such as racial discrimination, neighborhood safety, financial burdens, stigma, health literacy, and relationships with health professionals. She then used this patient-specific information to guide her engagement and supportive counseling of the patient and provided interested patients with the names and contact information for culturally-sensitive psychotherapists.

Patients in both interventions were asked by their DCM to try at least two educational materials, which were mailed after the initial telephone conversation. The educational materials include brochures (2004 versions), books, and DVDs/videotapes. The patient-centered intervention materials were culturally targeted and included a study calendar and a nondenominational depression prayer card for patients who described themselves as spiritual. Materials provided in each intervention are shown in Table 2[42-46]. The Black and Blue video/DVD [47] was innovative in featuring actual African-American patients rather than actors, targeting barriers to treatment for African Americans identified in previous research, and incorporating the viewpoints of African-American primary care physicians, mental health professionals, and clergy. The BRIDGE study calendar offered additional reinforcement on topics such as depression in the African-American community, substance abuse, safe and effective therapy and medication, spirituality, self-help, and suicide prevention.

**Table 2:** Patient education materials

	Standard intervention	Patient-centered intervention
**Communication material**		

**Book**		

How to Heal Depression [42]	X	

Chicken Soup for the African American Soul [43]		X

**Print media**		

Depression [44]	X	

Depression and African Americans [45]		X

Prayer Card		X

Real Men, Real Depression [44]	X	

Men and Depression^1 ^[44]		X

**Visual media**		

Coping with Symptoms of Depression, DVD or Video [46]	X	

Black and Blue, DVD or Video [47]		X

Calendar		X

^1^Distributed to men only

#### Data collection at baseline and over follow-up

In addition to the main outcome and process measures, baseline data were collected to describe the characteristics of study subjects and compare these characteristics between the intervention groups assigned by randomization. While the intervention status is the main predictor variable, other factors known to be predictors of depression (*e.g*., social support) and factors that might explain why the intervention did or did not work (*e.g*., clinician and patient attitudes regarding treatment) were collected. In general, we selected instruments that are brief, have been used successfully in primary care settings, and are reliable and valid in primary care clinicians and African Americans. We also collected detailed data on racial identity and other attitudes, beliefs, and experiences that might identify intra-ethnic group differences and help determine the study's external validity. We conducted telephone interviews to measure patients' depression status, attitudes regarding treatment, use of health services and experiences of care, and reactions to the interventions at six and 12 months of follow-up. Table 3 shows the variables collected from individual clinicians at baseline (pre-intervention), immediately after their in-person intervention contacts (post-intervention), and at the end of the study. Table 4 shows socio-demographic and behavioral variables, clinical measures, and health service utilization patterns collected from patients at baseline and over follow-up.

**Table 3:** Schedule of data collected from primary care clinicians in the BRIDGE study

Overall data collection	Pre intervention	Post intervention
Demographics (age, gender, race, ethnicity, place of birth, residency training, board certification status, practice experience)	X	

Specialty (internal medicine or family medicine)	X	

Previous communication skills CME training	X	X

Previous mental health CME training	X	X

Readiness to change behavior re: management of depression	X	

Knowledge, attitudes, self-efficacy re: managing depression	X	

Attitudes about race^1^	X	X

Self-reported communication and PDM style	X	

Job stress and satisfaction	X	

Self-efficacy in managing adherence problems, depression, and patients from socially and culturally diverse backgrounds	X	X

**Visit level data collection**		

Videotape with simulated patient	X	

Audiotapes with 5 to 10 depressed patients		X

Visit-specific satisfaction with each patient		X

Perceptions of patients' social and behavioral characteristics		X

Use/process evaluation of CD-ROM/workbook^2^		X

Rating of intervention effectiveness		X

^1^Explicit attitudes measured at baseline before enrollment visit; implicit attitudes measured only at end of study using the Implicit Association Test (IAT).

^2^Patient-centered intervention providers only

**Table 4:** Schedule of variables collected from patients in the BRIDGE study

Measurement/collection method	Enrollment visit	6 months	12 months	18 months
**Questionnaires**				

Sociodemographics	X		X	X
Age, sex, race/ethnicity, education, income, employment^1^, health insurance^1^, financial stress^¶^				

Attitudes, beliefs, and other behavioral measures	X	X	X	X
Trust in health professionals, preferred role in decision-making, depression treatment preferences, spirituality, respect, perceived involvement in care^2^, racial identity, social support, life events				

Health Status	X	X	X	X
Physical and mental, measured by MOS-SF12, CIDI^3 ^&ES-D, psychiatric co-morbidity^4^, disability days				
Healthcare Utilization	X	X	X	X
Mental healthcare utilization (receipt of antidepressant medication and/or counseling) General healthcare utilization (emergency room visits and hospitalizations)				
Healthcare Process	X	X	X	X
PDM with providers, visit-specific and overall satisfaction, satisfaction with case manager^5^, ratings of depression care management and intervention materials^6^				

**Audiotapes **(patient-provider communication)^7^	X			

CIDI = Composite International Diagnostic Interview, Depression Scale, CES-D = Center for Epidemiologic Studies Depression Scale, PDM = participatory decision making; ^1^measured at 12 and 18 months; ^2^measured only at 18 months; ^3^not measured at six months; ^4^Substance abuse, panic attacks, anxiety, and traumatic events; ^5^measured only at six and 12 months; ^6^measured only at 12 months only; ^7^collected at enrollment visit and after primary care provider intervention.

One modification to the original study protocol was made shortly after the 12-month follow-up was complete. Because the pattern of improvement differed between intervention groups at six months and 12 months of follow-up, a decision was made to extend follow-up to one additional interview at 18 months, and IRB approval was obtained to re-contact study patients. However, because outcomes assessors at 18 months were not blinded to intervention assignment, depression status at 12 months remains the study's primary outcome.

#### Main outcome measures and statistical analysis plan

Randomly assigned intervention group (standard versus patient-centered) is the main independent variable for intent-to-treat (ITT) analyses. Clinic site is a stratification variable for randomization and is expected to be balanced across intervention groups by design. The primary outcome variable is change in depression symptom severity, upon which we base the sample size evaluation to ensure proper statistical power. Descriptive statistics will be used to describe patient and clinician characteristics at baseline. The comparability of clinician characteristics between intervention groups will be determined for sociodemographic data as well as pre-intervention measures of training, attitudes, management of depression, and communication style. Comparability of patient characteristics between intervention groups will be assessed with regard to pre-intervention sociodemographics, depression level, health status measures, and other key variables. Variables found to be unbalanced between intervention groups will be adjusted for in all ITT analyses. Adherence to the intervention (*e.g*. number of intervention visits kept) will be considered as a covariate in subsidiary, on-treatment analyses.

The data on outcome variables fall into two broad categories: continuous variables, such as depressive symptom score (CES-D) [48] and functional status scores (MOS SF-12) [49], and dichotomous variables, such as depression remission status and receipt of guideline-concordant treatment. The primary ITT analyses are designed as regression modeling using patients as the unit of analysis, with each regression model structured to have a parameter designated for each visit where the outcome of the model was scheduled to be assessed, separately for each intervention group, so that the mean model is fully saturated for the intervention by visit parametrization. This parametrization allows estimation of mean differences observed within each intervention group betweeen baseline and a given follow-up visit for a continuous outcome, and odds ratios of having the outcome event at a given follow-up versus the baseline visit within each intervention group for a binary outcome, after adjusting for other covariates in the model. The evalution of patient-centered intervention effects will be conducted by testing the significance of difference of the adjusted mean differences, or the ratio of adjusted odds ratios, of a given follow-up versus the baseline visit between the intervention groups. The primary testing contrast is the baseline to 12-month change in outcomes between intervention groups; the models can easily accommodate additional data obtained by extending the follow-up to 18 months.

Site and clinician characteristics are included as covariates in all regression models to account for potential patient outcome clustering within clinical sites and/or clinicians. To account for longitudinal correlations between outcome assessed over time within patients, mixed effects modeling appraches with unstructured correlation matrix are used whenever possible. Robust estimation is used for statistical inferences. The likelihood approach underlying the proposed mixed effects modeling would produce valid statistical inferences with missing data if the missing mechanism is Missing at Random (MAR) and the proposed mean and correlation models are correct. We thus anchor our ITT analyses on mean models with fully saturated visit by intervention parametrization plus all the covariates as necessary, paired with an unstructured correlation model to develop valid statistical inferences under MAR. We then conduct sensitivity analyses through multiple imputation under plausible missing scenarios that are non-MAR to examine the robustness of our inferences under such situations.

#### Sample size considerations

One of the major hypotheses to be tested is that improvements in CES-D scores for depressed patients in the patient-centered intervention group will be greater than those in the standard intervention group. Previous studies of treatment of depression in primary care using similar interventions (excluding the patient-centered and cultural adaptations) have shown remission and symptom improvement differences between intervention and usual care groups of 20 to 30% [19,20]. Our resources and available recruitment pools allowed us to aim for 15 clinicians and 125 patients per group, with an average number of 8.3 patients per physician (cluster). By assuming a within-cluster or intra-class correlation of 0.1 [50] and 15% dropouts by the end of 12 months follow-up (reflected using the variance inflation factor), a correlation between the baseline and 12 month CES-D score of 0.5, a mean CES-D score of 30 points at baseline for both intervention groups, 25% score reduction (*i.e*., 7.5 points) by 12 months in the standard intervention group, and the common cross-sectional SD for CES-D of 10 points for both intervention groups at both visits, we would have 80% statistical power to detect a between-group difference of 3.6 points or more in improvement in mean CES-D score at one year using a two-sided test with Type I error of 0.05. That is, if we expect to see a 7.5 point improvement in mean CES-D score for the standard intervention group after one year, then we would have 80% power to detect a 11.1 points or greater improvement in mean CES-D score for the patient-centered intervention group after one year.

Ethics and Consent

The trial received approval from the Johns Hopkins and MedStar Health Institutional Review Boards. Informed written consent was obtained from all participating clinicians and patients. Subjects were free to withdraw from the study at any time, to refuse to answer any question, and to either stop audiotaping or to have audiotapes of any visit dropped from the study. None of the patient information was released to their clinician, health care organization, or any other party without the patients' permission. Phone contacts to locate the study subject did not suggest the content of the study. All study data were stored in locked file cabinets at Johns Hopkins and not the clinical sites. Personal identifiers were removed as soon as possible. Audiotape data were transferred onto CD-ROMs for coding purposes, and stored in locked files after identifiers were removed. A code key is kept in a separate location restricted to the principal investigator and project director. Each clinician received an incentive of $200 paid to them individually or to their organization and each patient received $25 for completing each interview.

### Baseline characteristics of study sample

#### Baseline characteristics of the clinicians

**Figure 2:**
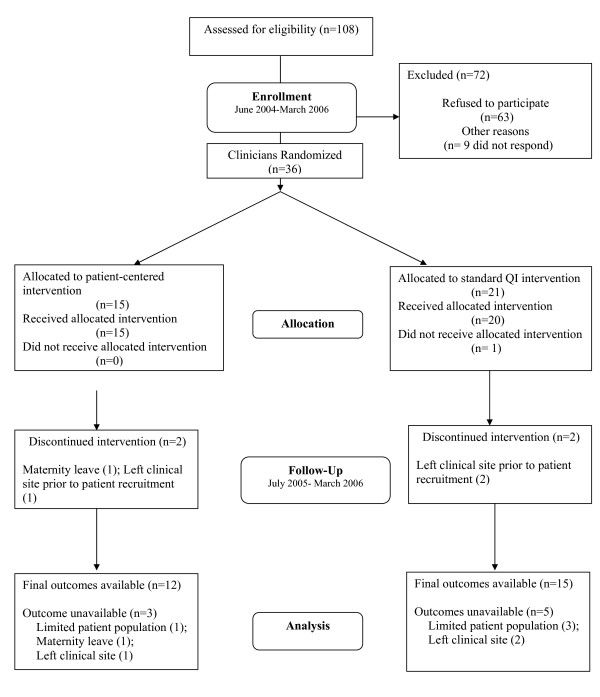
**BRIDGE study CONSORT flowchart for clinicians**.

Clinicians were enrolled between June 2004 and March 2006. We contacted 108 clinicians, of whom 72 were excluded; nine clinicians did not respond despite several phone calls, faxes, and emails from the study, and 63 clinicians refused to participate, citing lack of time or interest. Thirty-six clinicians were randomized to either the patient-centered intervention (n = 15) or the standard QI intervention (n = 21); one of these clinicians left the clinical site before baseline data collection and intervention delivery; eight clinicians were unable to contribute patients to the study (three left the site prior to patient recruitment; one went on maternity leave, and three were determined to have an inadequate sample of potentially eligible patients in their panel). (Figure 2) Characteristics of the 36 primary care clinicians recruited to the study are shown in Table 5. They were mostly general internists (58%) with a mean age of 42.4 years and mean practice experience of 6.9 years. Sixty-one percent were women, and they were ethnically diverse (28% African American, 17% Asian, and 47% White).

**Table 5:** BRIDGE Study: Demographic and baseline characteristics for 36 primary care clinicians

Characteristic	No. of PCPs (%)	Mean (standard deviation)
Age, years		42.4 (9.7)
Women	22 (61)	
Ethnicity		
African American	10 (28)	
Asian	6 (17)	
White	17 (47)	
Other	3 (8)	
Experience at current practice, years		6.9 (7.0)
Internal medicine	21 (58)	
Board certified	27 (75)	
CME in the past three years, hours		
Communication		6.2 (11.7)
Depression		5.0 (7.79)
Diversity		5.7 (20.12)
Definitely:		
Need to change way evaluate and manage MDD	3 (8)	
Make an effort to change way evaluate and manage MDD	7 (19)	
Likelihood of first-line treatment		
Very likely:		
Assess but not treatment	1 (3)	
Prescribe medication	18 (50)	
Counsel	6 (17)	
Refer to mental health specialist	14 (39)	
Very confident caring for:		
Socially disadvantaged	16 (44)	
Minority patients	19 (53)	
Very skilled in:		
Diagnosing major depression	9 (25)	
Managing simple antidepressant therapy (one med)	10 (29)	
Providing emotional support	5 (15)	
Following those in remission every six months	9 (25)	

#### Baseline characteristics of patients

**Figure 3:**
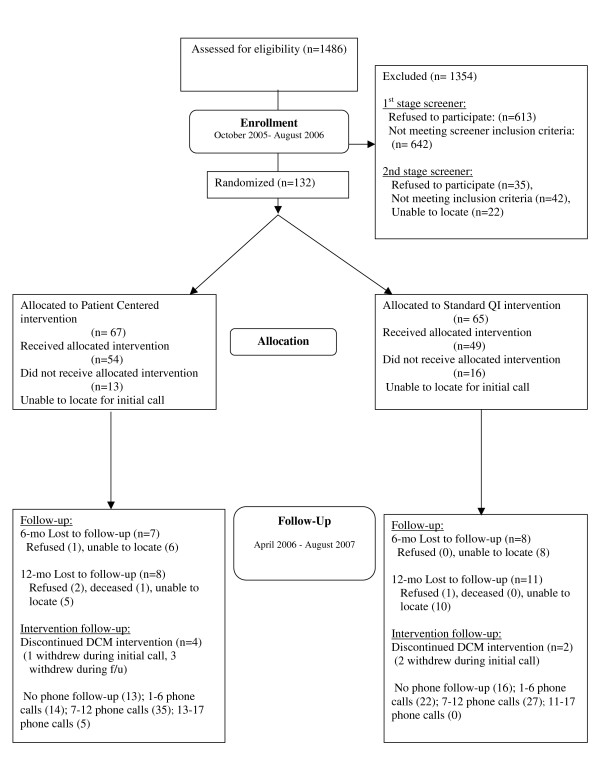
**BRIDGE study CONSORT flowchart for patients**.

Research assistants approached 1,486 patients for eligibility in waiting rooms between October 2005 and August 2006; 613 refused to complete in-person screening and 642 were determined to be ineligible by the first-stage screener (*e.g*. 457 were not depressed, 68 were positive for alcohol or drug abuse by the CAGE AID [51,52], and 46 were currently seeing a mental health specialist). Of the 231 patients eligible for the second-stage screen, conducted by telephone, 35 refused to complete it when called, 22 were unable to be reached to confirm their eligibility, and 42 were categorized as ineligible by the second-stage screen (CIDI). (Figure 3) Table 6 shows baseline characteristics of the 132 patients enrolled in the study. These patients are 46.4 years on average; 80% are women and 100% are African-American (4% also report being of Hispanic ethnicity). Forty-nine percent are high school graduates. The average annual household income is $41,393; however, 28% find it very difficult to pay their monthly bills. Fifty-eight percent are employed, and 89% have health insurance. The mean scores on the CES-D, MCS-12, and PCS-12 at baseline are 29.84, 36.18, and 42.12, respectively. Fifty-six percent of the sample reported having to take at least one disability day in the preceding two weeks.

**Table 6:** BRIDGE study: Baseline demographic and clinical characteristics for 132 African American patients

Characteristic	Number of patients (%)	Mean (standard deviation)
Age, years, mean (SD)		46.4 (11.1)
Gender, female	105 (79.6)	
Ethnicity		
Hispanic	5 (3.8)	
Non-Hispanic	127 (96.2)	
Marital status, married	36 (27.3)	
Education		
< High school graduate	12 (9.0)	
High school	65 (49.2)	
Some college	50 (37.9)	
College graduate	5 (3.8)	
Annual household income		$41,393 ($26,840)
Employed		
Full-time/part-time	77 (58.3)	
Other^1^	55 (41.7)	
Healthcare insurance Payer	117 (88.6)	
Medicaid	21 (18.0)	
Medicare	25 (21.4)	
Other	82 (70.1)	
CES-D score		29.84 (14.09)
MOS-SF-12, physical component		42.13 (13.2)
MOS-SF-12, mental component		36.18 (12.6)
Comorbid medical condition		
Diabetes	46 (34.9)	
Hypertension	72 (54.6)	
Trouble breathing	33 (25.0)	
Back problems	40 (30.3)	
Arthritis/rheumatism	56 (42.4)	
Had one or more disability days in last two weeks	74 (56.1)	
Taking antidepressant medication	101 (76.5)	
Treated for depression at most recent visit	65 (49.2)	

^1^Other = Retired, disabled, keeping house, attending school, or unemployed

## Discussion

Despite the proven efficacy of pharmacotherapy and psychotherapy for treatment of depression, many depressed primary care patients still do not receive adequate treatment. Moreover, African Americans and other ethnic minorities have lower rates of guideline-concordant treatment for depression than whites. Studies have identified patient-clinician communication and patient cultural beliefs, attitudes, and preferences as potential targets for interventions to improve outcomes and reduce disparities in care. Current state-of-the-art interventions still struggle to achieve one-year remission rates over 65% for patients with major depression [53]. While these interventions result in clinical improvement for ethnic minority patients, disparities in receipt of guideline-concordant care and functional outcomes are not eliminated [19].

This study compares the effectiveness of a patient-centered, culturally targeted adaptation with the standard conceptually-based QI intervention that was proven to be helpful for improving care for depression. The adapted intervention incorporates several successful features of previous QI interventions while also introducing novel elements. Both interventions use multifaceted approaches that include educational and epidemiological strategies to target intrinsic motivation and rational decision-making among health professionals [54]. The patient-centered adaptation aims to enhance clinicians' participatory decision-making skills and uses marketing strategies to adjust educational products and care management services to the needs of African Americans [54].

Limitations of the study should be discussed. The cluster design presents unique challenges with regard to comparability of groups (at the cluster and individual patient level), allocation concealment, maintenance of ITT principles, empty clusters, and participant switches from one cluster to another [55]. Several strategies have been recommended to overcome recruitment challenges: monitoring cluster guardian (*e.g*., practice leader) adherence to the study protocol before randomization of the cluster; including at least one patient participant before randomizing a cluster; and using recruiters who are blinded to participant allocation status. In this study, there was loss to follow-up among randomized clinicians before patient recruitment, which led to empty clusters (*e.g*., clinicians who did not contribute patients to the study) and failure to reach the recruitment target among patients, which may compromise the study's statistical power to detect differences in the primary outcome. Due to the staff resources available for recruitment and time frame needed for delivery of the clinician interventions, it was not feasible to delay randomization of clinicians until the first patient in each cluster was recruited. However, recruiters in this study were blinded to the allocation status of each cluster and its patients. With regard to analysis challenges, empty clusters and patients who switch from one cluster to another may lead to violation of the ITT principle. Recommendations include taking into account both clusters and individuals that withdraw or are lost to follow-up by using *ad hoc *missing data methods; keeping clusters as they were randomized, and using adjustment or propensity-score methods to deal with potential imbalances in both cluster and individual characteristics. In addition to these strategies, we will conduct sensitivity analyses to examine the potential impact of deviation from assumptions underlying such analytic approaches on the robustness of the study findings. Another design constraint is the limited ability to identify which aspects of the multi component interventions are most effective; we intend to conduct *post-hoc *analyses to determine whether the number of intervention contacts, use of intervention materials, and rating of intervention effectiveness by clinicians and patients are related to study outcomes.

Limitations of the interventions include the lack of booster exposures for clinicians and the reliance on telephone contacts and limited focus on enhancing access to psychotherapy for patients. These limitations were dictated by concerns about interference of the clinician interventions with clinical care and productivity; lack of office space for interventionists; and heterogeneous access to mental health specialists among patients with different health insurance coverage. Data collection and follow-up limitations include the reliance on self-report measures of process and health outcomes and relatively short follow-up time frame for assessment of functional outcomes from patients.

Still, the BRIDGE study addresses several limitations of previous studies. It includes under-resourced community-based practices and clinicians and African-American patients who have not been well-represented in previous clinical trials to improve depression care. Moreover, the patient-centered and culturally tailored approach to depression care is a relatively new one that has not been tested in most previous studies. The investigators found establishing strong relationships and aligning priorities with practice leadership early in the design of the study and throughout its implementation enhanced the success of recruitment at particular sites. Additionally, drawing on the strengths of site-specific administrative staff and electronic medical resources, when available, and adapting to the needs of clinicians and practices increased the efficiency and effectiveness of patient recruitment and acceptability and delivery of the interventions. The BRIDGE study will provide knowledge about how to recruit and enroll clinicians and patients into community-based participatory research programs; how to change clinicians' behaviors with regard to depression care in actual practice; and how to improve acceptability of treatments, quality of care, and depression outcomes for African Americans and other ethnic minorities with depression.

## Competing interests

The authors declare that they have no competing interests.

## Authors' contributions

LC, DF, DR, SL, AP, JG, GN, ES, and NW conceived of and designed the study. LC, DF, BG, DR, SL, and NW participated in the analysis and interpretation of data. NW provided statistical expertise. LC, BG, and NW drafted the article. All authors read and approved the final manuscript.
